# Chemical Composition and Agronomic Traits of *Allium sativum* and *Allium ampeloprasum* Leaves and Bulbs and Their Action against *Listeria monocytogenes* and Other Food Pathogens

**DOI:** 10.3390/foods11070995

**Published:** 2022-03-29

**Authors:** Flavio Polito, Giuseppe Amato, Lucia Caputo, Vincenzo De Feo, Florinda Fratianni, Vincenzo Candido, Filomena Nazzaro

**Affiliations:** 1 Department of Pharmacy, University of Salerno, Via Giovanni Paolo II, 132, 84084 Fisciano, Italy; fpolito@unisa.it (F.P.); lcaputo@unisa.it (L.C.); defeo@unisa.it (V.D.F.); 2 Institute of Food Science, National Research Council, Via Roma 64, 83100 Avellino, Italy; giuseppe.amato119@gmail.com (G.A.); fratianni@isa.cnr.it (F.F.); 3 Department of European and Mediterranean Culture, University of Basilicata, Via San Biagio, 75100 Matera, Italy; vincenzo.candido@unibas.it

**Keywords:** *Allium* *sativum*, *Allium ampeloprasum*, essential oil, biofilm, food pathogens

## Abstract

In this work, we aimed to study the chemical composition of the essential oils from bulbs and leaves of two cultivars of *Allium sativum* L. and two of *A. ampeloprasum* L. var. *holmense*. Moreover, we investigated their activity against four common bacterial strains responsible for food contamination (*Listeria monocytogenes*, *Escherichia coli*, *Acinetobacter baumannii*, and *Staphylococcus aureus*) by formation of biofilms. The susceptibility of bacterial biofilms was evaluated by crystal violet assay, whereas the metabolic changes occurring in the bacterial cells were ascertained through the MTT test. The essential oils were characterized by the presence of most characteristic components, although with different composition between the species and the cultivars. The essential oils inhibited the capacity of the pathogenic bacteria to form biofilms (up to 79.85 against *L. monocytogenes*) and/or acted on their cell metabolism (with inhibition of 68.57% and 68.89% against *L. monocytogenes* and *S.* *aureus*, respectively). The capacity of the essential oils to act against these foodborne bacteria could suggests further ideas for industrial applications and confirms the versatility of these essential oils as food preservatives.

## 1. Introduction

The term *Allium* identifies a very large genus of monocotyledonous plants, including about 700 plant species, organized into 15 subgenera and 72 sections [1]. The subgenus *Allium* is the largest, comprising approximately 280 species [2], 114 of which make up its largest section, *Allium* [3], which includes economically important species, such as garlic (*A. sativum* L.) and leek (*A. ampeloprasum* L.). Its first use is as a condiment, but it is also employed for therapeutic purposes due to the properties attributed to it jointly by scientific investigation and traditional medicine. Due to its widespread cultivation, *A. sativum* is almost ubiquitous, with origins in central Asia but quickly spreading in the Mediterranean basin and already known in ancient Egypt [4]. *A. ampeloprasum* is native to all countries bordering the Black Sea, as well as the Adriatic and Mediterranean Seas, North Africa; it is also present in Ethiopia, Uzbekistan, Iran, and Iraq.

*Allium* plants are generally perennial and herbaceous. Their prevalent biological form is bulbous geophyte (G bulb). Roots are fasciculated and coming out from the terminal part of the bulb. The stem is characterized by a bulbaceous hypogeal part (rarely rhizomatous or simple tuberous-type roots), the bulbs of which can be singular or numerous (aggregated); small, with an elongated oval shape; or large and globose and covered by a fibrous, reticulated, or smooth tunic surface. The epigeal part of the stem instead starts directly from the bulbs; some stems are fistulous, generally with a round section. At the base, the scape is wrapped in sheaths. Leaves are present in spirals, with an elongated, narrow, or enlarged shape but always flattened or almost cylindrical; in all cases, the length is preponderant over the width. These species have attracted human interest due to their flavor, taste, therapeutic properties, and ornamental value. For these reasons, they have been cultivated for thousands of years. Modern science confirmed that the plants of the genus *Allium* exhibit a wide variety of medicinal effects, such as defense against pathogens, prevention and treatment of cancer and cardiovascular disease, neuroprotection, hepatoprotection, and antifatigue effects [5,6,7,8,9,10,11].

*A. sativum* is one of the oldest cultivated species used in herbal medicine for therapeutic purposes in many cultures. Ancient medical texts documented medical applications of garlic. It has antihypertensive, anthelminthic, antioxidant, antithrombotic, antibiotic, antiseptic, and balsamic properties [12]. The species are differentiated into five cultivated vegetables, namely leek, elephant garlic, spring onion, kurrat, and Persian leek.

As a plant with multiple properties, *A. ampeloprasum* is classified as an edible officinal plant and exploited for its wide therapeutic and health properties. In fact, since ancient times, it has also been used in folk medicine to promote digestion and treat malfunctioning of the intestines. It reduces blood pressure, helps in dissolving kidney stones, prevents cramps and colds, helps to lower cholesterol, and can decongest the respiratory tract [13].

The essential oils (EOs) of *A. sativum* and *A. ampeloprasum* largely reflect the general composition of the oils obtained from plants of this subgenus. Some differences distinguish the two species regarding the presence and concentration of certain compounds. *A. sativum* contains a much more varied pool of sulfur compounds than *A. ampeloprasum*. The latter is characterized by the same main compounds (especially dimethyl sulfide) but has a much smaller variety of components. Over the past 50 years, intense research evaluated the biological activity of the EOs of the genus *Allium*. The organosulfides contribute to its use as an antioxidant [14,15,16,17]. Allicin contributes to the anti-inflammatory property, and it would seem to be a good candidate for the treatment of inflammation-related neurodegenerative diseases, such as Alzheimer’s [18,19,20,21,22]. Allicin and sulfur compounds derived from alliin metabolism have been shown to promote apoptosis in neoplastic cells [23]. Sulfur compounds can decrease the hepatic synthesis of cholesterol and the oxidation of LDL and HDL [24,25]. Allyl propyl disulfide, allicin, cysteine sulfoxide, and S-allyl cysteine decrease blood sugar, fasting cholesterol lipids [26,27], and cellular sensitivity to insulin [28]. Some components of these EOs showed effect on obesity [29] and inhibit platelet aggregation [30,31,32,33,34]. *Allium* EOs have antiviral [35], antiprotozoal [36,37], antifungal [38,39], and antibacterial [40,41,42,43] activities. 

In recent years, the rising occurrence of foodborne diseases has been correlated to an expansion of the presence, in foods, of some pathogenic bacteria, such as *Listeria monocytogenes*, *Escherichia coli*, *Pseudomonas aeruginosa*, and the emergent pathogen *Acinobacter baumannii*, often with the capacity to exhibit the multiple-drug resistance (MDR) phenotype [44]. Several bacterial strains, including *Listeria monocytogenes*, *Escherichia coli*, *Pseudomonas aeruginosa*, and *Acinobacter baumannii*, can produce biofilms, causing a serious problem for the food industry. This contamination can involve all stages of production, from harvesting and processing to storage [45,46,47]. The extracts of *A. sativum* exhibited clear evidence of antibacterial activity against different foodborne pathogenic bacteria [48]. Several papers reported the antibiofilm activity of *A. sativum*, but no studies reported antibiofilm activity exhibited by the EO of *A. ampeloprasum*. Caputo and colleagues reported the antibiofilm activity of extracts of two cultivars of *A. ampeloprasum* [49]. Thus, the utilization of *Allium* EOs can be of great importance in the food industry for the preservation of food from specific foodborne pathogens in all segments of the productive chain.

In the present work, we aimed to study the chemical composition of the EOs from two cultivars of *A. sativum* and two of *A. ampeloprasum* var. *holmense*, as well as their possible antibacterial activity against four pathogens of food interest—*Listeria monocytogenes*, *Acinetobacter baumannii*, *Escherichia coli*, and *Pseudomonas aeruginosa*—evaluating their capacity to inhibit formation and growth of biofilms and metabolism of bacterial cells.

## 2. Materials and Methods

### 2.1. Plant Material

Plants of two cultivars of *A. sativum*, cv ‘Rosso di Sulmona’ and cv ‘Rosso di Spagna’ and two cultivars of *A. ampeloprasum* var. *holmense,* cv ‘Contursi T.’ and cv ‘Irsinia’ were collected in May–June 2020. The cultivars were grown in an experimental field at Pontecagnano (Salerno province, Southern Italy,) on a previously ploughed and fertilized fine-texture soil. Cloves of all cultivars were planted in the middle of November 2019 with a spacing of 10 cm (*A. sativum*) or 20 cm (*A. ampeloprasum*) in rows spaced 50 cm apart in order to obtain densities of 20 and 10 plants per m^2^, for *A. sativum* and *A. ampeloprasum*, respectively. All cultivars were arranged in 5 m^2^ plots (2.0 m × 2.5 m) according to a randomized block design with three reps. Moreover, the normal agronomic practices of local garlic growers were followed. At harvest time, samples of 10 plants randomly taken from each plot were analyzed for the morphological traits reported in Table 1.

### 2.2. Extraction of Essential Oils

Samples were cleaned of residues of soil and other material and dried for about one week at room temperature. The plant material was divided into aerial parts and bulbs, which, separated and classified, were extracted with methanol at room temperature. This extraction was repeated three times, renewing the solvent. The extracts were then filtered using paper filters and freed of excess methanol using a rotavapor. Subsequently, the samples, with the minimum amount of methanol, were placed in a flask half-filled with water and subjected to steam distillation, as reported in the European Pharmacopoeia [50]. The obtained essential oils were solubilized in *n*-hexane, dried in a nitrogen atmosphere, and stored in amber vials in a refrigerator at 4 °C.

### 2.3. Composition of the Essential Oils

The EO composition was studied by GC and GC-MS. GC analyses were performed using a Perkin-Elmer Sigma-115 gas chromatograph equipped with FID and data handling processor. A HP-5 MS fused-silica capillary column (30 m × 0.25 mm i.d., 0.25 μm film thickness) was used, with the following operative conditions: column temperature: 40 °C, with 5 min initial hold, and then to 270 °C at 2 °C/min, 270 °C (20 min); injection mode splitless (1 μL of a 1:1000 *n*-hexane solution). Temperatures of injector and detector were 250 °C and 290 °C, respectively. Analysis was also performed with a fused silica HP Innowax polyethylenglycol capillary column (50 m × 0.20 mm i.d., 0.25 μm film thickness). In both cases, helium was used as carrier gas (1.0 mL/min). GC-MS analyses were carried out using an Agilent 6850 Ser. II apparatus, equipped with a fused silica DB-5 capillary column (30 m × 0.25 mm i.d., 0.33 μm film thickness), coupled to an Agilent Mass Selective Detector MSD 5973; ionization energy voltage 70 eV; electron multiplier voltage energy 2000 V. Mass spectra were scanned in the range 40–500 amu, scan time 5 scans/s. The GC conditions were as reported above; temperature of transfer line, 295 °C.

Most of the components were identified by comparing their Kovats indices (Ki) with those of the literature [51,52,53] and by analysis of the mass spectra compared to those of pure standards or to those reported in the NIST 02 and Wiley 257 mass libraries. The Kovats indices were determined related to a homologous series of *n*-alkanes (C10-C35), under the same operating conditions. For some compounds, the co-injection with standard samples confirmed the identification.

### 2.4. Antibacterial Properties of the Oils

#### Microorganisms and Culture Conditions

Gram-positive *Listeria monocytogenes* (ATCC 7644) and *Staphylococcus aureus* subsp. *aureus* (ATCC 25923) and Gram-negative *Acinetobacter baumannii (*ATCC 19606) and *Escherichia coli* (DSM 8579) were the tester bacterial strains. Bacteria were cultured in Luria–Bertani broth for 18 h at 37 °C (*A. baumannii* grew at 35 °C) and 80 rpm (Corning LSE, Pisa, Italy) for microbial analysis.

### 2.5. Minimal Inhibitory Concentration (MIC)

The MIC of each essential oil was evaluated through a resazurin microtiter-plate assay [54]. Multiwell plates were prepared in triplicate; then, they were incubated at 37 °C (35 °C for *A. baumannii*) for 24 h. The lowest concentration at which a colour change arose (from dark purple to colourless) determined the MIC value of each EO.

### 2.6. Biofilm Inhibitory Action of the EOs

The EOs capacity to influence the formation of bacterial biofilm was evaluated by the method of Caputo et al. [46] in flat-bottomed 96-well microtiter plates. Before the test, the overnight bacterial cultures were adjusted to 0.5 McFarland (1.5 × 10^7^ cells/mL, Densitometer cell density turbidity 0.3–15.0 McFarland, CAMLAB, Cambridge, United Kingdom) with fresh culture broth. Ten μL of the diluted cultures were placed in each well; then 10 μL/mL and 20 μL/mL of each EO and Luria-Bertani broth were added, for a final volume of 250 μL/well. Microplates were sealed with parafilm, to avoid the evaporation and incubated for 48 h at 37 °C (except for *A. baumannii,* incubated at 35 °C). Planktonic cells were removed and, subsequently, sterile PBS was used to wash the attached cells. Methanol (200 μL) was added to each well and kept for 15 min to fix the sessile cells. Methanol was discarded and the microplates were left to dry. The staining of the adhered cells was obtained addition of 200 μL of 2% *w*/*v* crystal violet solution, discarded after 20 min. Wells were lightly washed with sterile PBS and left to dry. Glacial acetic acid 20% *w*/*v* (200 μL) was added to obtain the release of the bound dye. The absorbance was measured at λ = 540 nm (Cary, Varian, Milano, Italy). The percent of adhesion was calculated respect to control; an inhibition of 0% was considered for cells without treatment. The tests were carried out in triplicate and the average results were taken for reproducibility.

### 2.7. Inhibition of Cell Metabolic Activity within the Biofilm 

Two concentrations (10 μL/mL and 20 μL/mL) of the EOs were assessed for their capacity to inhibit the metabolic activity of the bacterial cells through the 3-(4,5-dimethylthiazol-2-yl)-2,5-diphenyltetrazolium bromide (MTT) colorimetric method [55]. After 48 h total of incubation, planktonic cells were removed, and 150 μL of PBS and 30 μL of 0.3% of MTT (Sigma, Milano, Italy) was added, keeping microplates at 37 °C (*A. baumannii* was incubated at 35 °C). After 2 h, the MTT solution was expelled, and two washing steps were performed with 200 μL of sterile physiological solution; then, 200 μL of dimethyl sulfoxide (DMSO, Sigma, Milano, Italy) was added to allow for the dissolution of the formazan crystals that were measured at OD = 570 nm (Cary, Varian, Milano, Italy) after 2 h. 

### 2.8. Statistical Analysis

All assays were carried out in triplicate. Data of each experiment are expressed as the mean ± SD, and were statistically analyzed by two-way ANOVA, followed by Dunnet’s multiple comparisons test at a significance level of *p* *<* 0.05 using GraphPad Prism 6.0.

## 3. Results and Discussion

### 3.1. Morphological Traits of Bulbs and Cloves of Garlic Cultivars

As shown in Table 1, garlic bulbs and clove traits were significantly different among the tested cultivars. In particular, *A. ampeloprasum* showed larger bulbs and cloves than *A. sativum*. Conversely, the number of cloves per bulb was higher for *A. sativum*. Between *A. ampeloprasum* cultivars, cv ‘Contursi T.’ showed higher values for almost all traits, with the exception of the number of cloves per bulb, which remained significantly unchanged. Considering the *A. sativum* cultivars, mean weight and equatorial diameter of bulbs were significantly higher in ‘Rosso di Spagna’ compared to ‘Rosso di Sulmona’. Finally, the clove traits were not significantly different between the two cultivars.

### 3.2. Chemical Composition

The analysis of *A. ampeloprasum* var. *holmense* samples (Table 2) showed a quantitatively different composition between bulbs and aerial parts, even of the same cultivar. In the aerial parts of the cv. ‘Irsina’, 44 components were found, whereas in the bulbs, only 4 components were found. On the other hand, there is the opposite situation in the case of the cv ‘Contursi T.’, in which 10 components were found in the aerial parts, compared to 60 components found in the bulbs. The composition of the latter was particular, with a great variety of compounds generally present in low percentages. The composition reflects the data reported in the scarce literature available [33,56,57].

It must be emphasized that in many cases, the data on allicin do not correspond with what is reported in the literature. This is because the compound is very unstable and reactive and can rapidly decompose into other sulfur compounds. For this reason, compositional studies that have been characterized by different extraction or analysis techniques could report discordant data on the amount of allicin [58,59,60].

In all cases, the main compounds are sulfur compounds. Allicin appears to be the main component in the EOs from the aerial parts, with quantities that exceed 50% of the total—more precisely, 57.3% in the aerial parts of ‘Irsina’ and 53.1% in the aerial parts of ‘Contursi T.’.

The situation of bulbs is different, where allicin, despite being among the main compounds, is not the principal component. In fact, its percentages settle at 29.8% in the bulbs of ‘Irsina’ and 8.6% in the bulbs of ‘Contursi T.’.

The other main compounds differ depending on the plant. ‘Irsina’ contains high amounts of diallyl sulfide, which is the main component of the EO from the bulbs (42.5%), whereas the aerial parts contain 15.2% of this compound. The bulbs of ‘Contursi T.’ have propyl allyl sulfide as the main component (14.7%), whereas the aerial parts contain 34.4%.

These results agree with the literature, in particular with the studies by Satyal and colleagues (2017) [56] that showed that the majority of components of the EO of this species turn out to be the whole series of sulfur compounds, first of all diallyl disulfide, dipropyl disulfide, diallyl trisulfide, and dipropyl trisulfide.

The analysis of *A. sativum* samples showed (Table 3) a quantitatively richer composition as compared to that of the *A. ampeloprasum* samples. All the samples, except for the EO from the bulbs of *A. sativum* ‘Rosso di Sulmona’ and the aerial parts of *A. sativum* ‘Rosso di Spagna’, showed a very rich composition, in many cases exceeding 50 components, as in the case of the bulbs of the ‘Rosso di Spagna’ (77 components). The main components are the sulfur compounds. Allicin is once again the main component, with quantities exceeding 50%: 61.8% in ‘Rosso di Sulmona’ bulbs and 52,9% in the ‘Rosso Spagna’ bulbs. The aerial parts, on the other hand, contain lower quantities of allicin: 36.8% in ‘Rosso di Sulmona’ and 21.1% in cv ‘Rosso Spagna’. Diallyl disulfide appeared among the main components, becoming the most representative compound in the aerial parts of the ‘Rosso Spagna’ (48.5%). Other sulfur components were present: propyl allyl disulfide, contained in good amounts in the aerial parts of the ‘Rosso di Sulmona’ (30.6%). 

In this case, the results are in agreement with the literature [61,62], which reported a massive presence of sulfur compounds among which stand out dimethyl disulfide, diallyl disulfide, allyl methyl disulfide, propyl allyl disulfide, methyl propenyl disulfide, and diallyl trisulfide.

### 3.3. Biofilm Inhibitory Capacity of the EOs

The capacity of the EOs to inhibit bacterial biofilm formation and the metabolism of the bacterial cells within biofilm was assessed through crystal violet and MTT tests, respectively, using two concentrations—10 μL/mL and 20 μL/mL, amply lower than the minimal inhibitory concentration—calculated by the resazurin test and shown in Table 4 and Table 5.

A biofilm is an amassing of microorganisms on animate and inanimate surfaces with the support of extracellular polymeric substance (formed by proteins, polysaccharides, and nucleic acids), which has an important function in infection and bacterial resistance [61]. Biofilm formation facilitates such survival in the body [62]. Biofilms are considered important with respect to microbial survival and growth in the food industry. In fact, microbial growth in biofilms protects microorganisms against clean-up and sterilization and makes them more difficult to remove [63]. The antibacterial activity of the essential oil of *A. sativum* against many pathogenic bacteria, including antibiotic-resistant bacteria, such as the Shiga toxin-producing *E. coli* (STEC) [64] and the methicillin-resistant *Staphylococcus aureus* (MRSA), is well documented [65,66,67]. The EOs of the *Allium* variety bulbs were generally able to inhibit the formation of biofilm by the Gram-positive *L. monocytogenes*, which is an ubiquitous pathogen representing a major alarm for the food industry because it is an agent of the serious foodborne illness listeriosis. This bacterium can contaminate food products during different phases of processing, introduced to food industry environments by many means. *L. monocytogenes* may grow in biofilms, so it can be more protected against the environmental factors that tend to eradicate it. Some studies reported that the adherence to surfaces by *L. monocytogenes* is very important for its survival and persistence in food. When included in biofilm, this bacterium becomes more difficult to be removed. In recent decades, different approaches have been proposed to impede the adhesion of *L. monocytogenes*; however, they are difficult to be applied due to high costs and problems of resistance by the bacterium [68]. Thus, the world of natural biomolecules has been studied to find new solutions to limit the proliferation and virulence of *L. monocytogenes* during the steps concurring with food production [69]. From this point of view, therefore, our results seem very interesting. Because the crucial point of the growth and virulence of *L. monocytogenes* is its ability to adhere to surfaces (organic or inorganic), our data demonstrate that some of the extracts tested are capable of limiting such bacterial capacity. In fact, Table 6 indicates that the EO from the leaves of “Irsina” was capable of inhibiting up to 79.95% of the adhesive capacity of *L. monocytogenes;* such capacity was observed, although weaker, by testing 20 µL/mL of the EO from the bulbs of ‘Contursi T.’. The cultivars ‘Rosso di Sulmona’ and ‘Rosso di Spagna’ were capable of inhibiting the adhesion capacity of *L. monocytogenes*, with percentages of inhibition up to 64.11% and 61.22%, respectively. The action exhibited by these EOs vs. *L. monocytogenes* is in agreement with the literature. Jadhav and colleagues [70] and Sandasi and colleagues [71] showed that different EOs obtained from culinary and/or medicinal plants are capable of acting in reducing the attack of *L. monocytogenes* cells ab origine and therefore of influencing the formation of a subsequent biofilm by this microorganism. Recently, Somrani and colleagues [72] reported an excellent inhibitory biofilm activity by commercial EOs of *A. sativum* and *A. cepa*. However, biochemical variations of plants, which also affect their biological properties, can be related to the effects of genetic diversity, geographical origin, time of harvest, and the procedural methods used for the extraction [73].

In our experiments, the EOs from *A. sativum* were generally able to inhibit the formation of biofilms of all the bacterial strains tested. Furthermore, except in a few cases, all EOs were able to inhibit the formation of the biofilm of *A. baumanni*, a Gram-negative coccus known to cause nosocomial infections [74], where it provokes up to 30% mortality [75]. The EOs of *A. sativum* were also capable of inhibiting biofilm formation by *S. aureus*. In this case, the behavior exhibited by the EOs was different. In fact, the inhibitory action exhibited by the EO from the bulbs of ‘Rosso di Spagna’ was stronger than that of the EO from aerial parts (70.29% and 44.39%, respectively). Conversely, the EO of the aerial parts of ‘Rosso di Sulmona’ were more capable of inhibiting the *S. aureus* biofilm, with an inhibition value of 33.48% (with respect to 1.12% shown by the EO from bulbs, which was almost completely ineffective against *S. aureus*).

It is also important to emphasize the inhibitory efficacy exerted by the EOs vs. *E. coli*. In fact, at the highest concentration of EO used in the experiments, all the EOs of *A. sativum* proved capable of inhibiting, albeit with greater or lesser vigor, the biofilm of this bacterium, reaching inhibition percentages up to 54.09% (EO of the bulb of ‘Rosso di Spagna’). Our data disagree with those reported by Yang and colleagues. [76]. On the other hand, the cv ‘Rosso di Sulmona’, the bulbs of which contained more than double the allicin (61.8%), was slightly less effective in inhibiting the biofilm formed by this microorganism (41.20% inhibition). The two cultivars of *A. ampeloprasum* proved capable of inhibiting the formation of bacterial biofilms with varying effectiveness. The EO from the cv. “Irsina” proved to be more effective than the EO from the cv ‘Contursi T.’ in the sense that it was able to inhibit—more or less with the same effectiveness—the formation of the biofilms of the four bacteria. The EO obtained from the aerial parts of ‘Contursi T.’, although ineffective vs. *L. monocytogenes*, was able to achieve up to 81.88% inhibition of the *E. coli* biofilm and up to 73.47% of that formed by *S. aureus*. The EO obtained from the bulbs of ‘Contursi T.’ was ineffective vs. *A. baumannii* but managed to inhibit the biofilm of the other three pathogens, with inhibition percentages ranging between 25.39% (vs. *E. coli*) and 61.41% (vs. S. *aureus*). Few reports reported the antibacterial effects of the EOs from *A. ampeloprasum*. Methanolic extracts from bulbs and aerial parts of this species demonstrated biofilm-inhibitory activity against *L. monocytogenes*, *E. coli*, *P. aeruginosa*, and *S. aureus* [49].

#### Action of EOs against Bacterial Metabolism

Through the MTT test, the potential of EOs to inhibit the metabolism of bacterial cells present within the biofilm was also evaluated. The results are shown in Table 7. The EOs from both the aerial parts and bulbs of *A. ampeloprasum* were overall able to act on the metabolism of the microbial cells present within the biofilm. In the case of the tests carried out against *L. monocytogenes*, the results obtained with the EOs from ‘Irsina’ and ‘Contursi T.’ corroborated the already interesting data obtained by the crystal violet test. In fact, in this case, the EOs demonstrated an ability not only to limit the adhesive capacity of *L. monocytogenes* but also to affect, albeit more weakly, its metabolism. The EO from ‘Contursi T.’ showed an inhibitory effect of up to 25.28%; the EO from ‘Irsina’ was slightly stronger in inhibiting the metabolism of the bacterial cells within the biofilm, although such capacity was observed when we used 20 µL/mL The inhibitory activity of the EOs of ‘Rosso di Sulmona’ aerial parts was much more powerful, with an inhibitory effect on cell metabolism of up to 68.57%; a similar action was provided by the EOs of the ‘Rosso di Spagna’ aerial parts (60.20%). The EOs obtained from both the aerial and bulb parts of “Contursi T.” were extremely effective in inhibiting the metabolism of *A. baumannii* (89.47% and 81.14%, respectively). The EOs from ‘Irsina’ proved to be able to counteract the metabolic changes occurring to the cells within the biofilm. We also observed a good inhibitory effect against *E. coli*, with inhibition percentages never lower than 63.86% for the EO from ‘Contursi T.’. Instead, the EO from the bulbs of ‘Irsina’ were completely ineffective against *E. coli*, unlike the EO from the aerial parts, which, when tested at the highest concentration, resulted in an inhibition of 71.08% compared to the control. The effect on bacterial metabolism exerted by the EOs of *A. ampeloprasum* was instead more labile when tested against *L. monocytogenes* and *S. aureus*. However, bearing in mind that the action of the oils was particularly effective on the formation of the biofilm of these microorganisms, we can affirm that the two EOs of *A. ampeloprasum* tested turned out to be able to fight the pathogenicity of these four microbial strains, either by acting on the formation of the biofilm or by inhibiting those biochemical changes that affect the cells enveloped and protected by the biofilm and which determine the triggering of a series of biochemical events that lead the bacterium to prove itself more resistant, even to antibiotics [69]. This was also observed with the EOs of *A. sativum*, which, in some cases, in the face of an incisive biofilm-inhibitory activity, did not exhibit an equal activity on cellular metabolism. This was the case, for example, of the action exerted by the EOs vs. *E. coli*, as in the case of the EOs from ‘Rosso di Sulmona’. On the other hand, the EOs from aerial parts of ‘Rosso di Spagna’ did not show the same inhibitory capacity, being able to exert an inhibition of, at most, 18.27% and only at the highest dose tested.

The different effects of the four EOs confirmed once again that the EOs can act as antibiofilm agents, as amply demonstrated with other essential oils [77,78]. Our data show that the antibiofilm activity of these EOs is probably due to the ample presence of allicin and diallyl disulfide. These data are in agreement with the recent literature [79,80,81,82].

## 4. Conclusions

In this work, we showed that there is diversity in the chemical composition between the two species of A. *ampeloprasum* var. *holmense* and A. *sativum* and within the same species between the cultivars. The chemical compositions confirmed the presence of the main and most characteristic compounds as allicin and sulfur compounds, as reported in literature. These compounds were responsible for biological activities. The essential oils obtained, although differing in efficacy, demonstrated their capability to act against the formation of new biofilms, which is a key step in the increase in virulence of pathogenic bacteria, mainly for *L. monocytogenes*. Our results comfort us about the possibility of using these essential oils as potential preserving agents in food manufacturing, for instance, in the manufacturing of fermented meats, where the taste and smell of *Allium* EOs (both *A. sativum* and *A. ampeloprasum*) used as ingredients at the right concentrations do not have a negative effect from a sensorial point of view and can safeguard the products without affecting their quality. Moreover, from our data, it is possible hypothesize the use of these EOs both during the manufacturing processes and on the finished product; on this latter, they can be used as a food additive to maintain the biological properties described above. However, the EOs must be used mainly during the manufacturing process to avoid the formation of biofilms on the total product. In fact, if EOs were used only on the finished product, there would be an antibacterial action only on the external parts. The most promising EOs appear to be those extracted from aerial parts and bulbs of *A. amploprasum* ‘Irsina’ and from aerial parts of *A. sativum* ‘Rosso di Spagna’.

## Figures and Tables

**Table 1:** Morphological traits of bulbs and cloves of garlic cultivars.

Cultivars ^1^	Species	Bulb Skin Colour	Clove Skin Colour	Floral Stem	Bulb Mean Weight	Bulb Equatorial Diameter	Cloves per Bulb	Clove Mean Weight
					(g)	(mm)	(n.)	(g)
‘Rosso di Sulmona’	*A. sativum*	white	red	yes	41.2 (±0.8) b	48.1 (±0.9) b	11.8 (±0.9) a	3.1 (±0.7) a
‘Rosso di Spagna’	*A. sativum*	cream	red	yes	50.3 (±1.4) a	54.6 (±1.0) a	11.3 (±0.7) a	3.7 (±0.9) a
‘Irsina’	*A. ampeloprasum*	cream	l. brown ^2^	yes	68.1 (±0.4) b	75.1 (±0.9) b	5.3 (±0.6) a	11.4 (±0.2) b
‘Contursi T.’	*A. ampeloprasum*	cream	l. brown ^2^	yes	75.0 (±0.9) a	84.2 (±1.1) a	5.1 (±0.5) a	12.1 (±0.4) a

^1^ Means followed by the same letters in the same column and within each *Allium* species are not significantly (*p* ≤ 0.05) different. ^2^ l. brown = light brown.

**Table 2:** Chemical composition of EOs from *A. ampeloprasum* var. *holmense*, cultivars ‘Irsina’ and ‘Contursi T.’.

		%					
		‘Irsina’		‘Contursi T.’			
N.		Aerial Parts	Bulbs	Aerial Parts	Bulbs	RT	KI_a_
1	2,4-Dimethylhexane	-	22.3	-	-	5.0	758
2	3-Methylthiophene	-	3.4	-	-	7.4	788
3	2,2-bis (Methylthio)-1-propanol	-	-	1.7	-	9.2	812
4	2,6-Dimethylnonane	0.1	-	-	-	11.3	838
5	2,3,5,8-Tetramethyldecane	T	-	-	1.3	12.2	851
6	4-Methyl-1-undecene	0.1	-	-	0.3	12.3	852
7	Diallyl disulfide	-	-	0.1	0.2	12.9	860
8	2-Hydroxyethyl-disulfide	T	-	-	-	15.2	889
9	Borneol	0.1	-	-	-	15.5	893
10	Terpinen-4-ol	T	-	-	-	15.9	898
11	Tridecane	-	-	-	0.3	16.6	907
12	1,1-Thiobis-1-butine	-	-	-	0.3	16.8	910
13	Dimethyl sulfide	0.1	-	-	0.4	17.5	919
14	(Z)-Methyl propenyl disulfide	T	-	-	0.3	17.9	925
15	2,6,10-Trimethyldodecane	T	-	-	1.0	18.2	928
16	Dodecyl sulfide	-	-	-	0.1	18.5	932
17	Dodecyl-7-en disulfide	T	-	-	2.5	18.7	934
18	Dodecyl-8-en disulfide	-	-	-	0.4	18.9	937
19	Methyl octane	-	-	-	0.4	19.0	939
20	Carvacrol	4.1	-	-	-	19.2	941
21	*n*-Heptene	-	-	-	0.3	19.4	942
22	2-Methoxy-4-vinylphenol	-	-	-	1.1	20.0	950
23	Hexanal	0.1	-	-	0.5	20.6	959
24	(E)-Allyl propyl disulfide	0.2	-	-	0.2	21.0	964
25	(Z)- Allyl propyl disulfide	0.1	-	-	0.1	22.1	979
26	Hexanol	-	-	-	0.8	22.2	979
27	Octane	-	-	-	1.2	22.4	982
28	Decane disulfide	-	-	-	0.8	23.1	992
29	Geranyl isovalerate	-	-	-	0.3	23.3	994
30	Nonanal	0.1	-	-	2.8	23.5	997
31	Nonene	-	-	-	3.3	24.0	1000
32	Decene	0.3	-	-	6.1	24.4	1005
33	2,4-Bis(1,1-dimethyl-ethyl)-phenol	0.1	-	-	1.1	24.7	1009
34	2-Butyl-1-octanol	0.3	-	0.1	1.6	25.0	1013
35	Butyl octene	-	-	-	2.2	25.2	1017
36	*n*-Nonane	-	-	-	1.0	26.8	1038
37	(Z)-9-Ottadecene	0.1	-	-	-	26.5	1033
38	Propyl trisulfure	-	-	-	1.1	27.4	1046
39	1,3,5-Trithiane	-	-	-	2.1	27.8	1050
40	Undecane	-	-	-	0.8	28.4	1059
41	Undecene	-	-	-	0.9	28.6	1061
42	Methyl propenyl trisulfide	-	-	-	7.7	29.1	1068
43	Methyl 12-methyltridecanoate	0.8	-	0.3	0.5	29.5	1073
44	Methyl triacontanoate	-	-	-	0.7	29.6	1075
45	Ethyl 2-oxo-tetradecanoate	T	-	-	-	29.9	1079
46	Methyl pentadecanoate	0.7	-	-	-	30.8	1088
47	*trans*-Methyl -3-pentil-undadecanoate	0.1	-	-	-	31.2	1097
48	Propenyl trisulfide	0.1	-	3.2	2.8	31.8	1098
49	Propyl allyl disulfide	0.9	-	34.4	14.7	31.9	1100
50	Methyl 14-methyl-pentadecanoate	0.4	-	-	-	32.8	1114
51	Methyl (Z)- 9-esadecanoate	0.8	-	-	-	33.1	1116
52	Methyl 11-esadecanoate	0.9	-	-	-	33.4	1120
53	2-Hexyl-1-octanol	0.1	-	-	-	33.5	1122
54	Diallyl disulfide	15.2	42.5	-	-	33.8	1126
55	Propyl allyl trisulfide	0.2	-	-	-	34.0	1129
56	Methy 14-methyl-esadecanoate	0.4	-	-	-	34.9	1143
57	Methyl 2-Hexyl-cyclopropan-octanoate	0.2	-	-	-	35.0	1144
58	Methyl Heptadecanoate	0.4	-	-	0.5	35.5	1151
59	Methyl (Z)-9-octadecenoate	0.1	-	-	0.6	36.2	1160
60	Allicin	57.3	29.8	53.1	8.6	37.0	1171
61	Methyl allicin	7.0	-	3.2	-	37.3	1176
62	Diallyl trisulfide	2.6	-	-	1.0	37.6	1182
63	Methyl 8,11-ottadienoate	-	-	-	1.2	37.9	1185
64	Methyl 10-oxo-octadecanoate	-	-	-	0.6	38.1	1187
65	Methyl alliy trisulfide	-	-	-	0.2	38.3	1191
66	Methyl diallyl trisulfide	0.7	-	-	0.3	38.4	1192
67	Ethyl allyl trisulfide	-	-	-	0.1	39.3	1199
68	Ethyl diallyl trisulfide	1.9	-	1.5	2.9	39.4	1199
69	Vinyl diallyl trisulfide	0.1	-	-	2.7	40.8	1221
70	Propenyl trisulfide	0.2	-	-	1.1	41.3	1229
71	Heptadecan trisulfide	-	-	-	1.8	41.7	1235
72	Di-tert-dodecyl disulfide	T	-	-	-	42.0	1241
73	Octadecan trisulfide	-	-	-	0.2	42.4	1247
74	Pentadecan tetrasulfide	-	-	0.3	0.9	43.4	1262
75	Methyl esacosanoate	T	-	-	0.4	43.9	1269
76	Methyl 9,12-*epi*thio-9,11-octadecanoate	-	-	-	2.7	44.2	1274
77	Diallyl tetrasulfide	-	-	-	2.6	44.9	1284
78	Propyl allyl tetrasulfide	-	-	-	0.9	46.4	1300
79	Methyl tetracosanoate	-	-	-	1.1	46.8	1307
80	Propyl 3-(octadeciloxi)-oleate	-	-	-	0.5	47.1	1313
81	Propyl pentyl tetrasulfide	-	-	-	2.0	47.3	1315
82	Cyclo octasulfide	-	-	-	2.7	50.1	1360
	Total	96.9	98.0	97.9	97.8		

RT = retention time; KI = Kovats index on an HP5 MS capillary column; T = traces, less than 0.05%; - = absent.

**Table 3:** Chemical composition of the EOs of A. sativum, cultivars ‘Rosso di Sulmona’ and ‘Rosso di Spagna’.

		Rosso di Sulmona		Rosso di Spagna			
		%					
N.		Aerial Parts	Bulbs	Aerial Parts	Bulbs	RT	KI_a_
1	2,4-Dimethylhexane	-	1.7	17.1	0.1	5.0	757
2	3,31-Thiobis-1-propane	-	-	-	T	6.4	776
3	3-Methyl-thiophene	-	-	-	T	7.4	788
4	2,3-Dimethyl- thiophene	-	-	-	T	7.7	792
5	Methyl-2-propenyl-disulfide	-	-	-	T	8.1	796
6	α-Pinene	-	-	-	T	8.5	803
7	2,2-Bis (Methylthio)-1-propanol	-	-	-	0.6	9.2	812
8	(-)-β-Pinene	-	-	-	T	9.9	819
9	2,6-Dimethylnonane	-	-	-	T	11.2	837
10	D-Limonene	-	-	-	T	11.4	840
11	1,1-Dimetoxi-cyclohexane	-	-	-	T	12,2	850
12	2,3,5,8-Tetramethyl-decane	0.7	-	-	T	12.3	851
13	4-Methyl-1-undecene	-	-	-	T	12.4	853
14	Butyl propenyl sulfide	-	-	-	T	12.5	855
15	Diallyl disulfide	-	-	-	0.2	12.9	859
16	4-Etenyl-1,2-dimethyl-benzene	-	-	-	T	14.3	877
17	Allyl-1-propenyl sulfide	-	-	-	T	14.7	882
18	9-Hydroxyethyl-ethyl-disulfide	-	-	-	0.1	15.1	888
19	2- Hydroxyethyl- disulfide	0.2	-	-	-	15.2	889
20	Benzyl methyl sulphide	-	-	-	T	15.3	889
21	3,4-Dimethyl-thiophene	-	-	-	0.1	15.4	891
22	2-Ethyl-5-[(2-ethylbuthyl) thio]-thiophene	0.1	-	-	0.2	16.1	901
23	Bis(1,1-dimethylpropyl) -disulfide	-	-	-	0.1	16.4	905
24	Tridecane	0.4	-	-	-	16.6	907
25	1,1-Thiobis-1-butine	-	-	-	T	16.9	910
27	Dimethyl disulfide	0.2	-	-	0.1	17.6	921
28	(Z)-Methyl propenyl disulfide	2.9	-	-	T	17.9	925
29	(E)- Methyl propenyl disulfide	-	-	-	T	18.0	926
30	2,6,10-Trimethyl-dodecane	-	-	-	T	18.2	928
31	Dodecyl sulfide	-	-	-	T	18.5	931
32	Dodecyl-7-en disulfide	-	-	-	T	18.6	934
33	Dodecyl-8-en disulfide	-	-	-	T	18.9	937
34	2-Methoxy-4-vinylphenol	-	-	-	0.1	19.8	948
35	(E)-Allyl propyl disulfide	0.4	-	-	0.6	21.0	965
36	(Z)-Allyl propyl disulfide	3.9	-	-	0.3	22.1	979
37	Methyl 9-oxo-nonanoate	-	-	-	T	22.8	983
38	Decane disulphide	-	-	-	T	23.1	992
39	Geranyl isovalerate	-	-	-	T	23.3	994
40	Nonene	1.1	-	2.7	-	24	1000
41	Decene	1.3	-	0.4	-	24.2	1002
42	2,4-Bis(1,1-dimethyl-ethyl) -phenol	1.1	-	0.7	0.3	24.7	1009
43	2-Butyl-1-octanol	1.5	-	-	0.6	25	1013
44	(E)-9-Octadecene	0.2	-	-	T	26.5	1033
45	4-Methyl-1-undecene	0.5	-	-	0.1	26.5	1034
46	1,3,5-Trithiane	2.1	-	-	0.2	27.8	1051
47	Methyl propenyl trisulfide	-	-	0.6	3.4	28.8	1064
48	Methyl 12-methyl-tridecanoate	2.6	-	-	0.2	29.5	1073
49	Methyl triacontanoate	-	-	-	0.1	29.6	1074
50	Ethyl 2-oxo-tetradecanoate	-	-	-	T	30.0	1079
51	Methyl pentadecanoate	-	-	-	0.1	30.8	1091
52	Methyl 12-methyl-tetradecanoate	-	-	-	0.2	31.0	1093
53	Methyl *trans*-3-pentil-oxiran-undecanoate	-	-	-	0.3	31.6	1094
54	Propenyl trisulfide	0.8	-	-	0.2	31.8	1098
55	Propyl allyl disulfide	-	-	-	0.1	31.9	1099
56	Propyl allyl trisulphide	30.6	-	4.9	-	32.0	1100
57	Vinyl trisulphide	-	-	-	0.1	32.5	1108
58	Methyl 14-methyl-pentadecanoate	-	-	-	0.2	3.,8	1113
59	Methyl (Z)-9-esadecanoate	-	-	-	0.7	33.1	1116
60	Diallyl disulfide	-	34.5	48.5	12.6	33.7	1125
61	Methyl 2-hexyl-cyclopropanoctanoate	-	-	-	0.2	35.1	1144
62	Tridecan trisulfide	0.3	-	-	0.2	35.5	1151
63	Sulfide cyclicoctatomic	-	-	-	T	35.9	1156
64	Methyl (Z)-11-octadecenoate	-	-	-	T	36.2	1160
65	Allicin	36.8	61.8	21.1	52.9	36.9	1170
66	Methyl allicin	9.8	-	1.8	-	37.4	1177
67	Methyl 8,11-octadecadienoate	-	-	-	4.3	37.7	1182
68	Diallyl trisulfide	-	-	-	5.4	37.8	1183
69	Methyl diallyl trisulfide	0.4	-	-	0.8	39.0	1194
70	Ethyl diallyl trisulfide	0.1	-	-	5.1	39.6	1203
71	Vinyl diallyl trisulfide	-	-	-	0.2	40.8	1222
72	Heptadecan trisulfide	-	-	-	0.5	41.6	1234
73	Di-tert-dodecyl disulfide	0.2	-	-	0.8	42.0	1241
74	Octadecan trisulfide	-	-	-	1.7	42.5	1248
75	Tridecan tetrasulfide	-	-	-	0.1	43.1	1258
76	Pentadecan tetrasulfide	-	-	-	0.6	43.2	1262
77	Methyl exacosanoate	-	-	-	0.1	43.9	1269
78	Methyl 9,12-*epi*thio-9,11-octadecanoate	-	-	-	0.7	44.0	1270
79	Diallyl tetrasulfide	-	-	-	0.3	44.9	1282
80	Propyl allyl tetrasulfide	-	-	-	0.1	45.4	1292
81	Methyl triacontanoate	-	-	0.4	0.3	45.5	1294
82	Methyl tetracosanoate	-	-	-	0.1	46.8	1308
83	Propyl 3-octadeciloxi-oleate	-	-	-	0.4	47.1	1312
	Total	98.2	98.0	98.2	96.7		

RT = retention time; KI = Kovats Index on an HP5 MS capillary column; T = traces, less than 0.05%; - = absent.

**Table 4:** Minimal inhibitory concentration (µL/mL) of the EOS from cultivars of *A. ampeloprasum* var. *holmense* necessary to inhibit the growth of the pathogenic bacterial strains *Listeria monocytogenes*, *Escherichia coli*, *Acinetobacter baumannii*, and *Staphylococcus aureus*.

		*A. baumannii*	*E. coli*	*L. monocytogenes*	*S. aureus*
‘Irsina’	Aerial parts	30 ± 2	40 ^a^ ± 3	30 ± 3	30 ± 2
	Bulbs	30 ± 3	30 ^c^ ± 3	30 ± 2	30 ± 3
“Contursi T.”	Aerial parts	30 ± 3	28 ± 2	40 ± 3	28 ± 2
	Bulbs	40 ^b^ ± 2	35 ^a^ ± 3	30 ± 2	30 ± 2
Tetracycline		31 ± 1	24 ± 3	39 ± 2	38 ± 2

The experiments were performed in triplicate and reported as the mean (±SD). a: *p* < 0.1; b: *p* < 0.001; c: *p* < 0.0001 compared with the tetracycline used as control (ANOVA followed by Dunnett’s multiple comparison test).

**Table 5:** Minimal inhibitory concentration (µL/mL) of the EOs from two cultivars of *A. sativum* necessary to inhibit the growth of the pathogenic bacterial strains *Listeria monocytogenes*, *Escherichia coli*, *Acinetobacter baumannii*, and *Staphylococcus aureus*.

		*A. baumannii*	*E. coli*	*L. monocytogenes*	*S. aureus*
‘Rosso di Sulmona’	Aerial parts	30 ± 2	40 ^a^ ± 3	30 ± 3	40 ± 2
	Bulbs	30 ± 2	30 ^b^ ± 3	30 ± 3	40 ± 2
‘Rosso di Spagna’	Aerial parts	30 ± 4	30 ^b^ ± 2	30 ± 4	30 ± 2
	Bulbs	30 ± 2	35 ^a^ ± 2	30 ± 3	28 ± 3
Tetracycline		31 ± 1	24 ± 3	39 ± 2	38 ± 2

The experiments were performed in triplicate and reported as the mean (±SD). a: *p* < 0.1; b: *p* < 0.0001 compared with the tetracycline used as control (ANOVA followed by Dunnett’s multiple comparison test).

**Table 6:** Inhibitory activity of the EOs from the cultivars of *A. ampeloprasum* var. *holmense* and *A. sativum* on the biofilm formation capacity of four pathogenic strains.

		*A. baumannii*	*E. coli*	*L. monocytogenes*	*S. aureus*
‘Irsina’	Aerial parts 10 µL/mL	47.18 ^a^ ± 1.59	0	1.23 ± 0.18	0
	Aerial parts 20 µL/mL	72.68 ^a^ ± 1.42	22.58 ^a^ ± 0.93	79.85 ^a^ ± 1.05	57.97 ^a^ ± 1.11
	Bulbs 10 µL/mL	7.35 ^a^ ± 1.73	18.82 ^a^ ± 3.08	52.58 ^a^ ± 1.25	41.96 ^a^ ± 1.15
	Bulbs 20 µL/mL	52.83 ^a^ ± 1.14	45.95 ^a^ ± 0.81	63.24 ^a^ ± 1.72	50.17 ^a^ ± 0.82
‘Contursi T.’	Aerial parts 10 µL/mL	0	47.55 ^a^ ± 3.51	0	50.54 ^a^ ± 0.71
	Aerial parts 20 µL/mL	45.86 ^a^ ± 1.31	81.88 ^a^ ± 1.21	0	73.47 ^a^ ± 1.91
	Bulbs 10 µL/mL	0	0	20.65 ^a^ ± 3.2	11.11 ^a^ ± 1.8
	Bulbs 20 µL/mL	0	25.39 ^a^ ± 1.28	40.69 ^a^ ± 1,57	61.41 ^a^ ± 1.61
‘Rosso di Sulmona’	Aerial parts 10 µL/mL	0	12.21 ^a^ ± 1.91	46.06 ^a^ ± 1.83	25.52 ^a^ ± 1.59
	Aerial parts 20 µL/mL	61.76 ^a^ ± 3.17	36.31 ^a^ ± 1.47	64.11 ^a^ ± 0.74	33.48 ^a^ ± 2.16
	Bulbs 10 µL/mL	12.25 ^a^ ± 2.35	24.73 ^a^ ± 1.76	37.60 ^a^ ± 1.40	0
	Bulbs 20 µL/mL	48.55 ^a^ ± 1.52	41.20 ^a^ ± 3.37	42.03 ^a^ ± 0.54	1.12 ± 0.13
‘Rosso di Spagna’	Aerial parts 10 µL/mL	46.08 ^a^ ± 2.56	41.56 ^a^ ± 3.34	40.98 ^a^ ± 4.32	9.47 ^a^ ± 0.67
	Aerial parts 20 µL/mL	49.91 ^a^ ± 2.75	54.09 ^a^ ± 1.77	46.88 ^a^ ± 0.71	44.39 ^a^ ± 1.44
	Bulbs 10 µL/mL	26.62 ^a^ ± 3.02	0	44.84 ^a^ ± 4.64	15.25 ^a^ ± 0.38
	Bulbs 20 µL/mL	56.97 ^a^ ± 1.73	27.16 ^a^ ± 1.66	61.22 ^a^ ± 2.09	70.29 ^a^ ± 0.14

Results are expressed as percentages (mean ± SD) and calculated assuming the control (untreated bacteria, for which we assumed an inhibitory value = zero). a: *p* < 0.1 compared with the control (ANOVA followed by Dunnett’s multiple comparison test).

**Table 7:** Inhibitory activity of the EOs from the cultivars of *A. ampeloprasum* var. *holmense* and *A. sativum* on the cell metabolism of the pathogenic strains within the biofilm.

		*A. baumannii*	*E. coli*	*L. monocytogenes*	*S. aureus*
‘Irsina’	Aerial parts 10 µL/mL	0	18.26 ^a^ ± 1.52	0	0
	Aerial parts 20 µL/mL	30.13 ^a^ ± 0.28	71.08 ^a^ ± 2.47	31.43 ^a^ ± 0.31	55.99 ^a^ ± 1.1
	Bulbs 10 µL/mL	0	0	0	39.17 ^a^ ± 1.15
	Bulbs 20 µL/mL	14.48 ^a^ ± 0.13	0	30.25 ^a^ ± 0.68	47.81 ^a^ ± 0.82
‘Contursi T.’	Aerial parts 10 µL/mL	76.15 ^a^ ± 0.91	63.86 ^a^ ± 2.13	8.65 ^a^ ± 0.68	6.55 ^a^ ± 0.23
	Aerial parts 20 µL/mL	89.47 ^a^ ± 0.86	76.71 ^a^ ± 0.97	24.58 ^a^ ± 1.37	36.07 ^a^ ± 2.32
	Bulbs 10 µL/mL	69.28 ^a^ ± 1.37	65.08 ^a^ ± 0.99	8.46 ^a^ ± 0.55	7.97 ^a^ ± 0.94
	Bulbs 20 µL/ml	81.14 ^a^ ± 0.27	79.15 ^a^ ± 0.43	25.28 ^a^ ± 0.88	36.19 ^a^ ± 2.18
‘Rosso di Sulmona’	Aerial parts 10 µL/mL	0	0	20.67 ^a^ ± 1.67	22.07 ^a^ ± 1.59
	Aerial parts 20 µL/mL	16.94 ^a^ ± 1.13	0	68.57 ^a^ ± 0.89	30.34 ^a^ ± 2.16
	Bulbs 10 µL/mL	36.46 ^a^ ± 0.68	0	8.19 ^a^ ± 1.43	0
	Bulbs 20 µL/mL	65.16 ^a^ ± 0.85	0	11.13 ^a^ ± 1.65	0
‘Rosso di Spagna’	Aerial parts 10 µL/mL	6.11 ^a^ ± 0.67	0	6.58 ^a^ ± 2.14	5.19 ^a^ ± 0.67
	Aerial parts 20 µL/mL	25.75 ^a^ ± 0.69	18.27 ^a^ ± 0.66	60.20 ^a^ ± 2.06	41.77 ^a^ ± 1.44
	Bulbs 10 µL/mL	0	0	7.09 ^a^ ± 1.33	11.26 ^a^ ± 0.38
	Bulbs 20 µL/mL	45.88 ^a^ ± 1.50	4.27 ^a^ ± 0.69	13.61 ^a^ ± 1.65	68.89 ^a^ ± 1.45

Results are expressed as percentages (average ± SD) and calculated assuming the control (untreated bacteria, for which we assumed an inhibitory value= zero). a: *p* < 0.1 compared with the control (ANOVA followed by Dunnett’s multiple comparison test).

## Data Availability

The original contributions presented in the study are included in the article. Further inquiries can be directed to the corresponding author.
